# Substance and Behavioral Addictions, and Their Consequences among Vulnerable Populations

**DOI:** 10.3390/ijerph19106163

**Published:** 2022-05-19

**Authors:** Steve Sussman, Deborah Louise Sinclair

**Affiliations:** 1 Departments of Population and Public Health Sciences, and Psychology, and School of Social Work, University of Southern California, Los Angeles, CA 90032-3628, USA; 2 Department of Special Needs Education, Ghent University, 9000 Ghent, Belgium

“Vulnerable populations” in health behavior research, practice and policy is generally used to refer to groups that, due to their life circumstances, may require extra consideration, reasonable accommodation, and legitimized protection [1,2]. Other terms have been used, such as “special populations”, “at risk”, “disadvantaged”, “marginalized” and “underserved”, although any such term risks stigmatizing the group in question [3] (p. 198). How vulnerability is conceived of by researchers, practitioners, and policymakers determines who is considered vulnerable: defining its parameters too narrowly may exclude or stereotype group members, while too broadly extending it may trivialize the utility of the concept [4]. However, the term “vulnerable populations” may be ambiguously defined or undefined, leaving “who is vulnerable, why they are vulnerable, and what they are vulnerable to” open to interpretation [2] (p. 601). To attempt to clarify this term, one multifaceted definition defines a vulnerable population as an *outcome* of other variables, which concurrently represents a potentially *preventable*, *reversible process*, and is also a condition or *conditions* attached to a *specific group* [5]. A vulnerable population may refer to individuals with disabilities (physical or mental impairment that substantially limits major life activities), or individuals who represent socially or economically disadvantaged demographic populations, such as homeless individuals, rural families, foster children and those who have aged out of that system, as well as single parents, the elderly, racial/ethnic minorities, gender minorities, religious minorities, children with parents who are active-duty members of the Armed Forces, and veterans, among others. Vulnerability lies on a continuum of severity [6], and the intersections between various characteristics and circumstances may render some groups more vulnerable than others (i.e., a matter of degree, combinations of factors). Conceptions of vulnerability are important in public health practices and can shape public perceptions and responses.

In response to toxic life experiences, vulnerable persons may seek out alternative means of coping with stressors or may be subject to non-normative routes of socialization [7]. They may be at particular risk (vulnerable) of experiencing any number or type of substance or behavioral addictions [8]. Discourses on the etiology of addiction have historically emphasized the disproportionate vulnerability of specific groups. For example, children may be at heightened vulnerability for drug misuse in the context of familial or parental substance use (e.g., [9]), there are genetic and neurobiological markers of vulnerability that have been isolated [10,11], and unemployment, poverty, social position, available substances, and poor connection to one’s community [12,13] may confer heightened vulnerability. Contemporary models have distinguished between an individual-level oriented approach, characterized by one’s vulnerability versus resiliency, and an ecologically oriented approach rooted in the interactions between individuals nested within family systems and community-level factors [14]. As White [15] contends, “addiction is a disease of exposure—a collision between personal vulnerability and social opportunity. And that opportunity is often bred within psychological and social circumstances that made picking up again and again an attractive choice” (p. 1). One may attempt to cope with difficult life circumstances through entrenchment in an addiction, and entrenchment in the addiction may lead to increased vulnerability (downward drift [16]). The negative consequences resulting from entrapment by an addiction tend to interfere with aspects of one’s quality of life (QoL), and may diminish personal, family/social and community recovery capital, including the maximal performance of daily activities such as engaging in full-time work, the development of chronic impairment (e.g., peripheral neuropathy or vision loss with alcohol misuse), loss of energy, anxiety, depression, regret, lifestyle dissatisfaction, sense of meaninglessness, and loss of self-fulfilling leisure (e.g., [17,18,19]).

This Special Issue includes research articles and a review of vulnerable populations in the context of substance and behavioral addictions. As reflected in the set of articles, the call for papers defined ‘vulnerable populations’ broadly—as those uniquely impacted by the pushes (stresses) and pulls (seductions) of the lifestyle factors that facilitate addiction. Difficult, recurring life experiences may require legitimized assistance to prevent a (further) lowering of one’s QoL and reduction in recovery assets. Difficult life circumstances may consist of challenges at an intrapersonal level, microsocial level, or macrosocial/cultural level [16]. At the intrapersonal level, physical/medical challenges may define one as being part of a vulnerable population, and in need of extra assistance. One example is difficulties in coping with the effects of cardiovascular disease [20], which may involve seeking out alternative medicine remedies such as cannabis use (which tends to be under-regulated and can be misused), possibly due to a lack of access to other effective treatments. At a microsocial level, difficult experiences include adverse childhood experiences [21]. Parental substance use, mental illness, intimate partner violence, or parental divorce, as examples, may lead one to become vulnerable to outcomes including substance use, compulsive internet use, depression, or anxiety.

At the macrosocial/cultural level, blocked access to a quality education, living within low-resource communities (suffering poverty), impacts of marginalization, historical trauma, and discrimination may lead to addictions or other negatively consequential outcomes without extra assistance (e.g., rural African American females and tobacco use, ref. [22]; Native Hawaiian and other Pacific Islanders or NHPI and drug use, ref. [23]; migrants and ethnic minorities or MEM and drug use, ref. [24]; American Indian and Alaskan Natives or AIAN and drug use or problem gambling, ref. [25]). Certainly, being widely and recurrently stigmatized in the media and through governmental policies will lead one to become vulnerable to negative outcomes, regardless of one’s socioeconomic status. Examples include non-binary gender or transgender persons (TGD; ref. [26]) or coping with an established substance use disorder and being in the early stages of recovery [27]. Whether at an intrapersonal level, microsocial level, or macrosocial/cultural level, challenging conditions recurrently exerted on identified groups can lead to multiple or substitute substance and behavioral addictions.

Dynamic as well as static factors may confer ‘vulnerability’. Consequently, at least some sources of vulnerability may be modified or at least partially controlled [28]. That is, extra consideration, reasonable accommodation, and legitimized protection, may help arrest or remediate the conditions leading to vulnerability-related outcomes or attenuate the negative circumstances or consequences stemming from the generally unjust circumstances within which a group exists. Vulnerability-related outcomes include engagement in addictions, which may (further) impact one’s QoL but also may be a relatively dynamic factor, which can be addressed.

Addictive behavior is considered to be engaging in behavior for an appetitive effect, via achieving a period of satiation from an appetitive need (i.e., instinct, drive, neurobiological motive); then, after repeated appetitive behavior-satiation cycles, resulting in preoccupation over that behavior and a sense of loss of control regarding when and where, and how long, the behavior will be engaged in, leading to various undesired or negative consequences [16,29]. Substance and behavioral addictions may first provide addiction-specific subjective positive consequences, including feeling happier or less sad, feeling more energetic or temporarily quieted, experiencing feelings of accomplishment and security, feeling a sense of dominance or self-nurturance, enjoying new adventures or exploration to some extent, or a subjective experience of cognitive enrichment or decrease in obsessive thinking, as examples [30]. These effects may be sought out by a vulnerable population seeking to reduce the subjective impact of stressful daily events, experience the world differently, or alter their subjective sense of self. The effects of substance and behavioral addictions would not be a problem if there were no negative consequences (i.e., they might then simply be considered ‘passions’). Indeed, general involvement in behaviors such as the internet or smartphone use does not, in and of itself, reduce one’s QoL (e.g., see [31]), and may even increase the subjective QoL for some people (e.g., [32]). Conversely, intense preoccupation, loss of control, and undesired consequences (e.g., family complaints, poor work performance) would require remediation, even for electronic-media-related behavior.

Participating in such behaviors addictively tends to lower one’s QoL, such as leading to negative social reactions from others, unusual thinking, negative emotional outcomes, possibly financial problems, legal consequences, and medical issues (due to injuries or addiction-related sedentary behavior). Further, stigma is a consequence that operates regarding socially disapproved addictions, or possibly socially tolerated addictions at their extremes (e.g., the extremes of shopaholism), and may play a role in worsening the conditions among vulnerable populations through social castigation, self-rejection, or feelings of loneliness [33]. The remediation of diminished QoL likely demands individual-level or group-level treatment (e.g., practice of mindfulness, support persons or groups) that helps one cherish what one has not lost and relish what one gets back (e.g., [34,35,36]). However, some vulnerable populations may lack access to various treatment avenues that demand insurance or other payments.

Importantly, the remediation of diminished QoL among vulnerable groups, compounded by experiencing an addiction, likely demands environmental-level, systemic changes, involving policy enactment and the enforcement and provision of social services. For example, the cessation of substance use may lead to improvements in one’s QoL, but recovery takes time, and life options may not change much for several years (i.e., it may take time for “recovery capital” to accumulate; ref. [37,38,39]). One might consider the introduction of means to improve one’s QoL prior to achieving abstinence. As an example, vulnerable populations may need immediate assistance to obtain reasonable housing, access to food, and a safe social climate. There may be a need for psychiatric assistance. (See [38,40] regarding means to rebuild recovery capital.) However, one may experience substitute addictions once one abstains from drug misuse (e.g., other drugs such as tobacco, or various behavioral addictions), which could continue to negatively impacting one’s QoL (e.g., [27,41]) and depleting recovery capital. Thus, there may be a need to monitor for substitute addictions.

Researchers and practitioners are urged to reflect on ‘vulnerability’ in the context of addictive behaviors. Taken together, this Special Issue foregrounds that only addressing a particular addiction (e.g., entering a treatment facility for alcohol use disorder) would not alter the system-level status quo. Rather, means to enhance horizontal and vertical mobility [42], enrich social support networks (e.g., with recovery center alumni), and address individual variations in stigmatic conditions [33,43] are sorely needed to address the conditions and processes that give rise to or maintain vulnerability. 
